# Dynamic Changes in Cortical Activation Patterns During Incremental Load Among Athletes of Different Sports Types

**DOI:** 10.1002/brb3.71631

**Published:** 2026-07-24

**Authors:** Zhi Liu, Luxiang Cui, Xiaoqi Lu, Shengting Zhao, Yangyang Dong

**Affiliations:** ^1^ Graduate School Harbin Sport University Harbin China; ^2^ College of Sports Training Shenyang Sports University Shenyang China; ^3^ Kunming Tongren Hospital Kunming Medical University Kunming China; ^4^ Kunming Medical University Kunming China

**Keywords:** athletic specialization, brain functional connectivity, functional near‐infrared spectroscopy, motor‐related cortical regulatory response, open‐skill exercise, progressive fatigue

## Abstract

**Purpose:**

This study aimed to investigate cortical functional changes during progressive fatigue among athletes from different training backgrounds to elucidate distinct neural modulation patterns associated with various exercise types.

**Method:**

Ninety‐six male participants were recruited and assigned to four groups (*n* = 24 per group): the open‐skill training group (OTG), endurance training group (ETG), resistance training group (RTG), and control group (CG) comprising individuals without specialized training.

**Finding:**

Maximal oxygen uptake test results showed that the OTG and ETG exhibited significantly higher values than the RTG and CG. Heart rate variability (HRV) analysis revealed that the OTG demonstrated superior autonomic regulation compared with the other three groups, whereas both ETG and RTG showed higher HRV than the CG. In the motor cortex (MC), oxygenated hemoglobin (HbO) concentration was significantly higher in the OTG than in the RTG and CG and higher in the ETG compared with the CG. Total hemoglobin (HbT) in the MC was greater in the OTG than in the ETG, RTG, and CG and higher in the ETG compared with the CG. In the prefrontal cortex (PFC), HbO was higher in the OTG than in the ETG, RTG, and CG, whereas both the ETG and RTG showed higher values than the CG. Similarly, the HbT of PFC in the OTG was greater than in the ETG and CG, with ETG and RTG both higher than CG. Functional connectivity (FC) between the PFC and MC was stronger in the OTG, ETG, and RTG than in the CG. Across all groups, HbO, HbT, and FC increased with rising perceived exertion and declined at maximal fatigue.

**Conclusion:**

This cross‐sectional study reveals that a long‐term specialized training background is associated with distinct central nervous system phenotypes. Open‐skill athletes showed higher cortical‐autonomic coordination and cognitive–motor integration under progressive fatigue, which is consistent with differences in neurovascular characteristics and resource allocation patterns.

## Graphical abstract

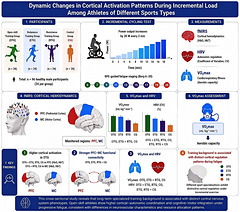

**Abbreviations**
ACC: anterior cingulate cortex
ANOVA: analysis of variance
ANS: autonomic nervous system
BDNF: brain‐derived neurotrophic factor
CAN: central autonomic network
CG: control group
CNS: central nervous system
CV: coefficient of variation
ETG: endurance training group
FC: functional connectivity
fNIRS: functional near‐infrared spectroscopy
HbO: oxygenated hemoglobin
HbT: hemoglobin total
HRV: heart rate variability
IGF‐1: insulin‐like growth factor‐1
M1: primary motor cortex
MC: motor cortex
OTG: open‐skill training group
PFC: prefrontal cortex
PMA: premotor area
ROIs: regions of interest
RPE: rating of perceived exertion scale
RTG: resistance training group
S1: primary sensory cortex
VEGF: vascular endothelial growth factor
VO_2_
: oxygen consumption
VO_2max_
: maximum oxygen uptake

## 1 Introduction

Fatigue is a core factor that influences athletic performance and training adaptation. It is essentially a multi‐level state of physiological and neural regulation imbalance. Fatigue includes not only the accumulation of peripheral muscle energy depletion but also the dynamic regulation by the central nervous system (CNS) in response to exercise intensity and body condition (Taylor et al. 2016; Ament and Verkerke 2009). Early research mainly focused on physiological changes at the muscle level, such as energy depletion, lactate accumulation, H^+^ imbalance, and increased free radicals, leading to the view of fatigue as a “muscle fatigue” phenomenon (Debold and Westerblad 2024). However, increasing evidence suggests that exercise fatigue is not merely a peripheral phenomenon but is also closely related to changes in the function of the CNS. Studies have found that with the increase in exercise intensity, both the prefrontal cortex (PFC) and primary motor cortex (MC) (M1) show increases in blood oxygen levels and neural activation, reflecting the CNS's “compensatory regulation” to counter peripheral fatigue (McMorris et al. 2018). However, when this compensatory regulation gradually becomes exhausted or imbalanced and the compensatory cortical activation can no longer be maintained, it manifests as a reduction in motor cortical output efficiency accompanied by weakened spinal efferent drive, ultimately limiting the performance of exercise capacity (Ament and Verkerke 2009). Therefore, uncovering the dynamic response characteristics of the CNS to progressive fatigue is crucial for understanding the neural basis of athletic performance and optimizing training strategies.

The CNS's regulation of exercise fatigue can be viewed as a dynamic process involving drive, compensation, and inhibition (Yu et al. 2025). At the beginning of exercise, the CNS enhances the MC's drive to spinal motor neurons, increasing the recruitment rate and firing frequency of motor units to ensure precise muscle control (Sale 1987). As exercise continues, especially under high‐intensity contractions, muscle fibers gradually fatigue, and the CNS compensates by increasing drive (Green 1997; Boccia et al. 2024; Liu et al. 2025). At this stage, subjective effort perception significantly increases.

When the compensatory mechanisms become difficult to sustain, imbalances in neurotransmitter metabolism (such as serotonin and dopamine) and enhanced inhibitory feedback signals from fatigued muscles lead to decreased excitability of the MC and restricted motor neuron firing, ultimately resulting in reduced central regulatory capacity and a decline in force production (Taylor et al. 2016; Amann et al. 2022). This process depends on the coordinated activity of distributed brain networks. The primary MC (M1) issues motor commands, the premotor and supplementary motor areas (premotor area [PMA] and SMA) participate in movement preparation and coordination, the PFC and anterior cingulate cortex (ACC) regulate cognitive effort and executive monitoring, whereas the primary sensory cortex (S1) and thalamus integrate sensory feedback from muscles and joints (Bhattacharjee et al. 2021; Yamaguchi et al. 2020). Thus, exercise fatigue is not only a result of physiological failure but also a proactive regulation and strategic adaptation formed by the CNS through the integration of multiple layers of information.

Long‐term specialized training induces different CNS adaptations in athletes, shaping their patterns of energy metabolism, motor control, and cognitive involvement (Kitazawa et al. 2021; Slattery et al. 2015). Endurance athletes, due to prolonged low‐intensity, sustained exercise stimuli, develop enhanced sensorimotor integration and automatic control abilities. This results in a lowered threshold for MC excitability and more stable coupling between sensory feedback and motor output (Taubert et al. 2015; Wieland et al. 2022). Meanwhile, the thalamocortical loop and spinal reflex activity are strengthened, forming an energy‐efficient motor regulation pattern characterized by automated control (Haeufle et al. 2020; Spooner and Wilson 2022). Strength athletes, on the other hand, develop higher cortical recruitment efficiency and corticospinal conduction capabilities under high‐intensity, short‐duration explosive loads, with synchronized motor unit recruitment and significantly increased strength output rate (Balshaw et al. 2016). Open‐skill training athletes, exposed to multiple stimuli from cognition, decision‐making, and movement in complex environments, exhibit more flexible coordination between the PFC, MC, cerebellum, and spinal cord (Heilmann et al. 2022; Yarrow et al. 2009). These athletes possess strong strength and reaction capabilities while maintaining stability in movement execution and strategy adjustment under fatigue conditions (Dambroz et al. 2022). Although previous studies have focused on fatigue differences between different types of athletes, most have concentrated on postexercise comparisons. There remains a lack of in‐depth understanding of the dynamic mechanisms of CNS involvement in fatigue development during exercise. Specifically, the brain activation and functional connectivity (FC) characteristics of athletes from different disciplines under the same subjective fatigue level have yet to be clarified, which may partially limit a comprehensive understanding of fatigue mechanisms and the refinement of individualized training strategies.

To address this, the current study employs incremental VO_2max_ testing to induce progressive fatigue, coupled with the rating of perceived exertion (RPE) scale to define different stages of fatigue. Using functional near‐infrared spectroscopy (fNIRS), brain cortical hemodynamic changes during exercise are dynamically monitored, allowing for a systematic comparison of brain region activation characteristics and FC patterns in athletes from different disciplines under the same fatigue condition. Compared to traditional electroencephalography, fNIRS offers higher resistance to movement artifacts and better spatial resolution, providing reliable brain function indicators in real‐world exercise scenarios.

Given that the present study employed a cross‐sectional design, the association between training type and neural phenotype may have been influenced by confounding factors, such as aerobic capacity, training load, metabolic phenotype, and self‐selection bias; therefore, the causal direction between training type and central adaptation cannot yet be established. This study aimed to characterize the differences in CNS regulation during progressive fatigue among individuals with different training backgrounds, thereby providing preliminary scientific evidence for subsequent longitudinal intervention studies and investigations into the neural mechanisms of exercise fatigue. We hypothesized that during incremental exercise, when athletes were at the same RPE level, individuals with different training backgrounds would exhibit significant differences in cortical activation intensity and FC patterns.

## 2 Materials and Methods

### 2.1 Study Participants

This study was a cross‐sectional investigation involving a total of 96 male participants who met the inclusion criteria (Supporting Information Appendix 1). According to their respective sports disciplines, participants were divided into four groups: the open‐skill training group (OTG), mainly consisting of basketball athletes (*n* = 24, 24 males); the endurance training group (ETG), mainly consisting of long‐distance runners (*n* = 24, 24 males); the resistance training group (RTG), mainly consisting of weightlifters (*n* = 24, 24 males); and the control group (CG), mainly consisting of individuals without specialized training (*n* = 24, 24 males).

Participants in this study were required to meet the following criteria: (1) age between 19 and 26 years; (2) a minimum of 3 years of continuous specialized training, with at least 10 h of training per week. The CG consisted of individuals with no specialized training experience; (3) all participants passed routine medical examinations and exercise risk assessments and were able to safely complete a VO_2max_ incremental load test; and (4) well‐controlled blood pressure within the past 2 years. For normotensive participants: resting systolic blood pressure <120 mmHg and diastolic blood pressure <80 mmHg. For participants with a history of hypertension: blood pressure controlled to <130/80 mmHg after medication treatment, with a stable medication regimen maintained for the past 3 months; (5) no history of neurological or psychiatric disorders, head trauma, or epilepsy. Additionally, participants had not taken any medications or supplements in the past 4 weeks that could affect central nervous or cardiovascular function (e.g., beta‐blockers and stimulants); (6) no history of lower limb musculoskeletal injuries or rehabilitation treatments in the past 3 months; and (7) for the fNIRS testing, participants’ hairstyles and scalp conditions were required not to interfere with the collection and processing of optical signals.

This study was approved by the Ethics Committee of Harbin Sports University (approval number: 2025035). All participants signed written informed consent forms after receiving sufficient information, and the study strictly adhered to the ethical principles outlined in the Declaration of Helsinki. The basic characteristics of the participants are presented in Table 1.

**TABLE 1:** Basic characteristics of participants.

	OTG	ETG	RTG	CG	*p* value
Age (years)	22.42 ± 2.67	23.08 ± 2.28	22.25 ± 2.11	22.38 ± 2.34	
Height (cm)	175.29 ± 6.52	175.33 ± 8.36	173.71 ± 7.54	176.17 ± 5.84	
Weight (kg)	65.3 ± 9.38	66.67 ± 9.63	66.74 ± 7.19	67.38 ± 6.08	0.73
BMI (kg/m^2^)	21.15 ± 1.95	21.58 ± 1.8	22.11 ± 1.77	21.69 ± 1.32	0.56
Training years (years)	5.28 ± 1.37	5.4 ± 1.36	5.02 ± 1.15		0.79
Weekly training time (h)	12.28 ± 1.37	12.23 ± 1.28	11.96 ± 1.17		0.81

Abbreviations: BMI, body mass index; CG, control group; ETG, endurance training group; OTG, open‐skill training group; RTG, resistance training group.

Data are presented as mean ± SD. Comparisons among the four groups were conducted using one‐way analysis of variance (ANOVA). Where normality or homogeneity of variance was violated, data were natural‐log transformed; if assumptions remained unmet, nonparametric tests were applied. There were no significant differences among groups in age, height, weight, body mass index (BMI), training years, or weekly training time (all *p* > 0.05). *n* = 24 per group (total *N* = 96).

### 2.2 Experimental Procedure

In the formal experiment, all participants are required to wear loose and comfortable sportswear, arrive at the laboratory in advance to familiarize themselves with the testing procedure, and undergo the experiment test. This study employed a single‐blind design. Prior to data collection, all participants were assigned randomized identification codes to anonymize both personal information and group allocation. As participants were aware of their own training history and sports specialization, participant blinding was not feasible. However, researchers responsible for signal preprocessing, data extraction, and statistical analyses remained blinded to group allocation throughout the entire data analysis process, thereby minimizing observer bias and interpretation bias. Participants complete a VO_2max_ incremental exercise test on a stationary cycle ergometer while undergoing fNIRS and heart rate monitoring. During the test, researchers record the participants’ subjective rating of RPE scores every 2 min.

To minimize external interference, participants are required to avoid strenuous exercise 24 h before the test; they must fast for 4 h prior to the test and refrain from engaging in strenuous physical activity in the 24 h preceding the evaluation. Additionally, participants must avoid alcohol and caffeine intake within 12 h of the test and ensure 7–9 h of sleep. All tests are conducted in the same laboratory, with the room temperature controlled between 22°C and 24°C, relative humidity maintained between 40% and 60%, and the environment kept quiet to avoid external light or noise disturbances, ensuring consistency in the testing environment. Each participant's test is performed by the same trained evaluator to reduce measurement errors caused by operational differences.

### 2.3 Measurement

#### 2.3.1 Maximal Oxygen Uptake Test

VO_2max_ is measured using the aerobic capacity (Metalyzer 3B, Cortex Biophysik GmbH, Germany) during a standardized incremental load test on a stationary cycle ergometer. The cycle ergometer is used to effectively eliminate any performance bias, allowing a better reflection of the dynamic changes in brain cortical involvement across different regions during the incremental exercise process (Brümmer et al. 2011). The test starts with a 2‐min warm‐up at a 30 W load, followed by a 20 W increase every 2 min. The cadence is maintained at ≥60 revolutions per minute according to a metronome and a cadence display. The test continues until the participant reaches the termination criteria for VO_2max_. The termination criteria are as follows: (1) a plateau in oxygen uptake, (2) the participant reports significant fatigue (e.g., general weakness, chest tightness, difficulty breathing, and dizziness) and requests to stop, (3) the respiratory exchange ratio exceeds 1.0, (4) the participant is unable to maintain ≥60 rpm even with verbal encouragement, and (5) the heart rate reaches 85%–90% of the predicted maximum heart rate (calculated by the formula: 208–0.7 × age). The highest oxygen uptake value (VO_2_) recorded during the test is defined as the VO_2peak_.

#### 2.3.2 Functional Near‐Infrared Brain Imaging Test

**FIGURE 1:**
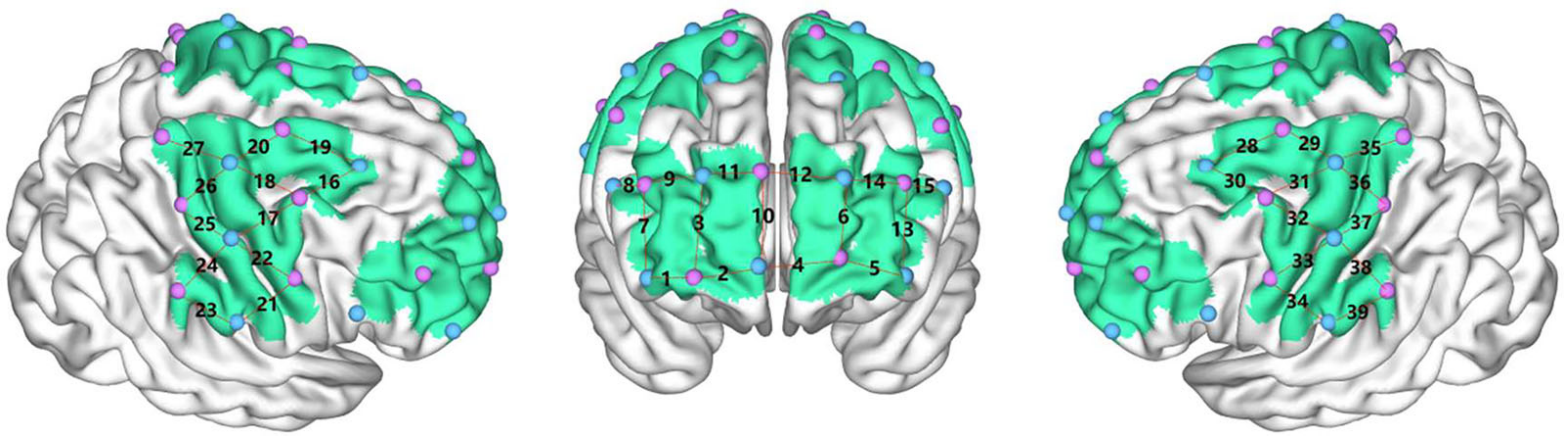
The configuration of the fNIRS probe: detectors (pink dots), light sources (blue dots), and channels (numbers).

fNIRS data are collected using a multichannel portable Nirsmart system (NirScan‐3000C, Danyang Huichuang Medical Equipment Co. Ltd., China). The system uses dual‐wavelength near‐infrared light sources (730 and 850 nm) and has a sampling frequency of 11 Hz. The electrode cap used in the experiment is designed according to the international 10/20 electrode placement system and is available in three sizes: large, medium, and small. The appropriate size is selected based on the participant's head circumference. A black sweatband is placed above the probe to block environmental light interference. During setup, the Cz point is used as a reference for positioning, and visual calibration is performed along the sagittal plane to ensure accurate placement. Before optode placement, the researcher followed a standardized procedure (parting the hair at a 45° angle, inserting the optode perpendicularly, and confirming that the scalp‐contact indicator light turned green). After the cap was fitted, signal quality across all channels was inspected, and invalid channels were excluded. All procedures were performed by the same trained researcher to minimize inter‐operator variability. Each participant uses a probe configuration consisting of 16 emitters and 16 detectors, forming 39 channels with a fixed distance of 30 mm between emitters and detectors (Lee et al. 2019). This channel layout covers the PFC and MC regions, allowing for the effective monitoring of dynamic changes in oxygenated hemoglobin (HbO) concentrations during the exercise. The brain regions of interest (ROIs) are divided into two main areas: the PFC and the motor‐related cortex. The motor‐related cortex includes the SMA, M1, PMA, and primary sensory cortex (S1). The channels monitoring the PFC include (CH1, CH2, CH3, CH4, CH5, CH6, CH7, CH8, CH9, CH10, CH11, CH12, CH13, CH14, and CH15), and the MC channels include (CH16, CH17, CH18, CH19, CH20, CH21, CH22, CH23, CH24, CH25, CH26, CH27, CH28, CH29, CH30, CH31, CH32, CH33, CH34, CH35, CH36, CH37, CH38, and CH39) (Supporting Information Appendix 2) (Figure 1).

#### 2.3.3 Perceived Exertion Scale and Heart Rate Monitoring

During the VO_2max_ incremental load test on the stationary cycle ergometer, participants’ subjective exercise perception is continuously assessed using the Borg 6–20 rating of RPE scale. The study selectively focused on nine stages with RPE values of 6, 7, 9, 11, 13, 15, 17, 19, and 20 for data analysis. Every 2 min, a trained evaluator asks the participant about their perceived exertion and records the RPE value to reflect the subjective fatigue level at different load stages.

This study employed the Borg 6–20 scale to continuously assess participants’ perceived exertion during the VO_2max_ incremental exercise test, and data analysis was performed at nine selected stages corresponding to RPE values of 6, 7, 9, 11, 13, 15, 17, 19, and 20. As a subjective perceptual indicator, RPE interpretation may be influenced by training background, exercise familiarity, and individual differences in perceptual calibration. Previous studies have shown that individuals with different training statuses may report divergent RPE values under the same physiological load. For example, endurance‐trained individuals, due to long‐term adaptation to aerobic exercise stimuli, may report lower RPE at the same blood lactate concentration, whereas resistance‐trained individuals, being relatively unfamiliar with cycle ergometer testing protocols, may overestimate their perceived exertion (Jurasz et al. 2022). In addition, large‐scale cross‐sectional studies have confirmed that RPE is modulated by multiple factors, including age, sex, testing modality, and exercise duration, and exhibits a complex interaction with VO_2max_ levels (Liu et al. 2026; He et al. 2026). Therefore, in this study, RPE was regarded as an “anchor for subjective fatigue stages” rather than an absolute indicator of equivalent physiological state.

#### 2.3.4 Heart Rate Monitoring

Participants’ heart rate was continuously monitored in real time using a Polar H10 device (Polar Electro, Kempele, Finland), and heart rate data were recorded synchronously with RPE and power output. Heart rate monitoring was conducted continuously throughout the entire testing procedure to accurately capture dynamic changes in exercise load and fatigue status. All measurements were performed by the same evaluator to reduce inter‐operator error.

In time‐domain analysis of heart rate variability (HRV), the coefficient of variation (CV) is an important index used to quantify the relative variability of heart rate data. Its calculation formula is $\left(\mathrm{CVHR}=\frac{\mathrm{SDHR}}{\bar{\mathrm{HR}}}\right)$. A higher CV indicates greater flexibility and adaptive capacity of the autonomic nervous system (Hillebrand et al. 2014; Pham et al. 2021).

Although most studies commonly use RMSSD and similar indices to assess parasympathetic activity, this study selected CV primarily because it better reflects the dispersion and stability of individual responses during training adaptation. Compared with RMSSD, which mainly reflects short‐term vagal activity and recovery status, CV is a dimensionless metric that is more suitable for comparing response variability across individuals and training conditions, thereby providing a more intuitive assessment of consistency in training adaptation.

The CV is less commonly used as an index of HRV, with most studies favoring time‐domain measures, such as RMSSD, to assess parasympathetic activity. In this study, CV was chosen because it reflects both inter‐ and intraindividual variability and stability of responses (Vivodtzev and Taylor 2021; Bellenger et al. 2016). Unlike RMSSD, which captures short‐term vagal modulation, CV is better suited to describing consistency and variability in physiological responses during training adaptation, providing a clearer view of interindividual differences across interventions (Ranieri et al. 2025). As a dimensionless metric, CV reduces the influence of absolute value differences and facilitates comparisons across individuals or measures (Gaspar et al. 2026). RMSSD, in contrast, is more sensitive to transient parasympathetic changes and is commonly used for monitoring recovery and fatigue (Vivodtzev and Taylor 2021; Bellenger et al. 2016). Thus, RMSSD is more appropriate for day‐to‐day training monitoring, whereas CV is more suitable for evaluating variability in responses to interventions.

### 2.4 Statistical Analysis

Statistical analysis is conducted using SPSS 27.0. Data visualization is performed with GraphPad Prism 9 software. For continuous quantitative variables, the Shapiro–Wilk test is first used to assess the normality of the data, and the Levene median test is applied to test the assumption of homogeneity of variance. If at least one of these conditions is not met, the data are transformed to a logarithmic scale (natural logarithm) to ensure normal distribution and/or homogeneity of variance. If these conditions are still not met after transformation, nonparametric tests are used for data analysis. The results are presented as descriptive statistics. One‐way ANOVA is used to compare baseline characteristics, VO_2max_ results, and HRV results between groups. Two‐way ANOVA is employed to compare the following indicators: the HbO and hemoglobin total (HbT) values in motor‐related cortical regions and PFC at different fatigue states during the VO_2max_ test, as well as the changes in brain FC at different fatigue states. Post hoc pairwise comparisons are conducted using the Bonferroni method. All tests are two‐tailed, and a *p* value ≤0.05 is considered statistically significant.

## 3 Results

### 3.1 Maximal Oxygen Uptake Test and HRV Results

**FIGURE 2:**
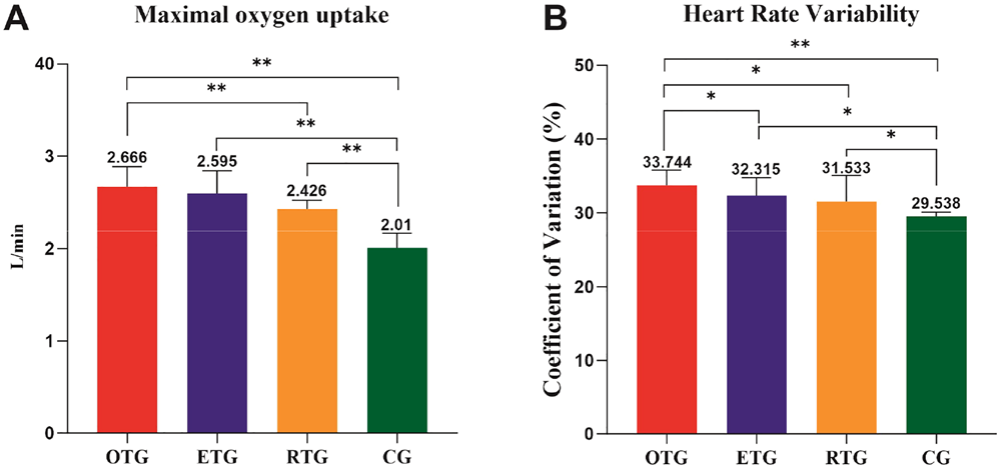
Cardiorespiratory fitness and autonomic function across training groups. (A) Maximal oxygen uptake (VO_2max_) and (B) heart rate variability (HRV, coefficient of variation) were compared among the open‐skill training group (OTG), endurance training group (ETG), resistance training group (RTG), and control group (CG). Group differences were analyzed using one‐way ANOVA followed by Bonferroni post hoc pairwise comparisons. For VO_2max_: *F* = 5.809, *p* = 0.001, *η* ^2^ = 0.159. OTG and ETG were significantly higher than RTG and CG (*p* < 0.05); RTG was significantly higher than CG (*p* < 0.05); and no significant difference was observed between OTG and ETG (*p* > 0.05). For HRV: *F* = 12.92, *p* = 0.001, *η* ^2^ = 0.296. OTG was significantly higher than ETG, RTG, and CG (*p* < 0.05); ETG and RTG were significantly higher than CG (*p* < 0.05). Data are presented as mean ± SD (*n* = 24 per group). *p < 0.05; **p < 0.005 (Bonferroni‐corrected).

In Figure 2A, a one‐way ANOVA revealed significant differences in VO_2max_ values among the four groups (*F* = 5.809, *p* = 0.001). The results showed that the VO_2max_ of the OTG was significantly higher than that of the RTG and the CG, but no significant difference was observed between the OTG and the ETG (*p* > 0.05). Similarly, the VO_2max_ of the ETG was significantly higher than that of the RTG and the CG. The VO_2max_ of the RTG was significantly higher than that of the CG (*p* < 0.05).

In Figure 2B, a one‐way ANOVA showed significant differences in HRV results among the four groups (*F* = 12.92, *p* = 0.001). The HRV of the OTG was significantly higher than that of the ETG, RTG, and CG, with statistical significance (*p* < 0.05). Similarly, the HRV of the ETG and RTG was significantly higher than that of the CG (*p* < 0.05).

### 3.2 Changes in HbO Concentration in the MC During the VO_2max_ Test

**FIGURE 3:**
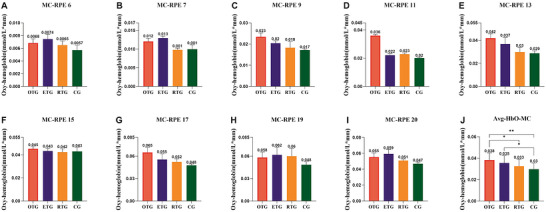
Dynamic changes in oxygenated hemoglobin (HbO) concentration in the motor cortex (MC) during incremental exercise. (A–I) Comparisons of mean HbO concentrations at respective rating of perceived exertion (RPE) stages: RPE 6 (A), 7 (B), 9 (C), 11 (D), 13 (E), 15 (F), 17 (G), 19 (H), and 20 (I). (J) Overall mean HbO concentration collapsed across all nine RPE stages. Statistical analysis was performed using two‐way repeated measures ANOVA (group × time) with Bonferroni post hoc tests. Group × time interaction: not significant (*p* > 0.05). Main effect of group: *F*(3, 92) = 7.445, *p* = 0.001, *η* ^2^ = 0.026. Pairwise comparisons: OTG > RTG (*p* = 0.017) and CG (*p* = 0.001); ETG > CG (*p* = 0.014); and no significant differences were observed between OTG versus ETG, ETG versus RTG, or RTG versus CG (*p* > 0.05). Main effect of time: *F*(8, 736) = 92.99, *p* = 0.001, *η* ^2^ = 0.473. HbO levels at RPE Stages 6–9 (RPE 15–20) were significantly higher than Stages 1–5 (RPE 6–13) (*p* < 0.05). Data are presented as mean ± SD (*n* = 24 per group). *p < 0.05; **p < 0.005 (Bonferroni‐corrected). CG, control group; ETG, endurance training group; OTG, open‐skill training group; RTG, resistance training group.

According to the two‐way ANOVA results of HbO concentration in the MC (Figure 3), as shown in Figure 3A–I, there were no significant differences in the mean HbO concentrations across RPE stages among groups (*p* > 0.05). However, the overall mean HbO concentration in the MC showed a significant group effect (*F*_group_ = 7.445, *P*_group_ = 0.001, *η* ^2^ = 0.026). This partial *η* ^2^ indicates a small‐to‐moderate effect size (between Cohen's benchmarks of 0.01 and 0.06), suggesting that training background explains 2.6% of the total variance in MC HbO. Pairwise comparisons using the Bonferroni correction revealed that the OTG exhibited significantly higher HbO levels than both the RTG (*p* = 0.017) and CG (*p* = 0.001) (*p* < 0.05). No significant difference was observed between OTG and ETG (*p* > 0.05). The ETG showed significantly higher HbO levels than the CG (*p* = 0.014, *p* < 0.05), whereas no significant differences were found between ETG and RTG or between RTG and CG (*p* > 0.05). In addition, a significant time effect was observed for HbO concentration in the MC across fatigue stages during the VO_2max_ test (*F*_time_ = 92.99, *P*_time_ = 0.001, *η* ^2^ = 0.473), with all four groups showing significantly higher HbO levels during Stages 6–9 (RPE 15–20) compared with Stages 1–5 (RPE 6–13) (*p* < 0.05).

### 3.3 Changes in HbT Concentration in the MC During the VO_2max_ Test

**FIGURE 4:**
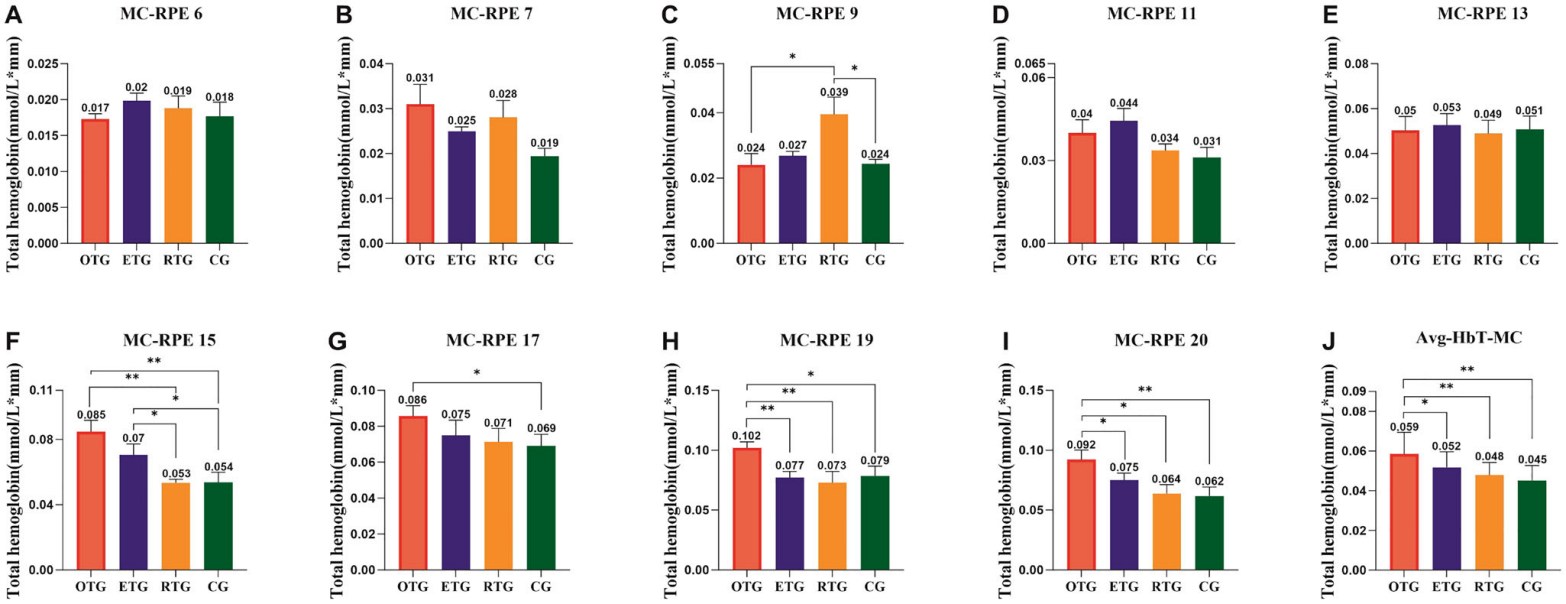
Dynamic changes in total hemoglobin (HbT) concentration in the motor cortex (MC) during incremental exercise. (A–I) Comparisons of mean HbT concentrations at respective RPE stages (RPE 6–20) among the four groups. (J) Overall mean HbT concentration across all fatigue stages. Statistical analysis: two‐way repeated measures ANOVA (group × time) with Bonferroni post hoc tests. Group × time interaction: Significant pairwise differences at specific stages were as follows: RPE 9 (C), RTG > OTG (*p* = 0.044) and CG (*p* = 0.05); RPE 15 (F), OTG > RTG (*p* = 0.001) and CG (*p* = 0.001), ETG > RTG (*p* = 0.027) and CG (*p* = 0.029); RPE 17 (G), OTG > CG (*p* = 0.031); RPE 19 (H), OTG > ETG (*p* = 0.001), RTG (*p* = 0.001), and CG (*p* = 0.002); RPE 20 (I), OTG > ETG (*p* = 0.025), RTG (*p* = 0.001), and CG (*p* = 0.001). Main effect of group: OTG > ETG (*p* = 0.001), RTG (*p* = 0.049), and CG (*p* = 0.001). Main effect of time: Stages 5–9 (RPE 13–20) were significantly higher than Stages 1–4 (RPE 6–11) (*p* < 0.05). Data are presented as mean ± SD (*n* = 24 per group). *p < 0.05; **p < 0.005 (Bonferroni‐corrected). CG, control group; ETG, endurance training group; OTG, open‐skill training group; RPE, rating of perceived exertion; RTG, resistance training group.

According to the two‐way ANOVA results for HbT concentration in the MC (Figure 4), as shown in Figure 4A–I, there were significant group‐by‐time interaction effects during the VO_2max_ test (*F*_group×time_ = 1.947, *P*_group×time_ = 0.004, *η* ^2^ = 0.053). Specifically, as illustrated in Figure 4C, the HbT concentration in the MC of the RTG was significantly higher than that of the OTG (*p* = 0.044) and CG (*p* = 0.05). In Figure 4F, the OTG exhibited significantly higher HbT levels than the RTG (*p* = 0.001) and CG (*p* = 0.001), whereas the ETG also showed significantly higher HbT concentrations than the RTG (*p* = 0.027) and CG (*p* = 0.029). In Figure 4G, the OTG displayed significantly higher HbT values than the CG (*p* = 0.031). In Figure 4H, the OTG demonstrated significantly higher HbT concentrations than the ETG (*p* = 0.001), RTG (*p* = 0.001), and CG (*p* = 0.002). Similarly, in Figure 4I, the OTG showed significantly higher HbT levels than the ETG (*p* = 0.025), RTG (*p* = 0.001), and CG (*p* = 0.001) (*p* < 0.05). The overall mean HbT concentration in the MC also showed a significant group effect (*F*_group_ = 10.433, *P*_group_ = 0.001, *η* ^2^ = 0.036), corresponding to a small‐to‐moderate effect size. Pairwise comparisons using the Bonferroni correction indicated that the OTG exhibited significantly higher HbT concentrations than the ETG (*p* = 0.001), RTG (*p* = 0.049), and CG (*p* = 0.001) (*p* < 0.05). In addition, a significant time effect was observed for HbT concentration across fatigue stages during the VO_2max_ test (*F*_time_ = 79.526, *P*_time_ = 0.001, *η* ^2^ = 0.435), with all four groups showing significantly higher HbT concentrations during Stages 5–9 (RPE 13–20) compared with Stages 1–4 (RPE 6–11) (*p* < 0.05).

### 3.4 Changes in HbO Concentration in the PFC During the VO_2max_ Test

**FIGURE 5:**
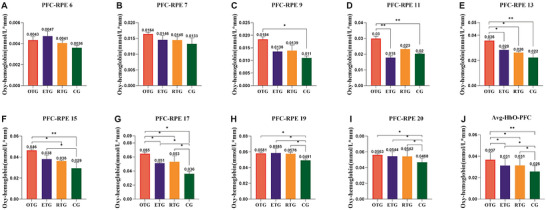
Dynamic changes in oxygenated hemoglobin (HbO) concentration in the prefrontal cortex (PFC) during incremental exercise. (A–I) Comparisons of mean HbO concentrations at respective RPE stages among the four groups. (J) Overall mean HbO concentration across all fatigue stages. Statistical analysis: two‐way repeated measures ANOVA (group × time) with Bonferroni post hoc tests. Group × time interaction: *F*(24, 736) = 2.464, *p* = 0.001, *η* ^2^ = 0.067. Significant pairwise differences at specific stages: RPE 9 (C), OTG > CG (*p* = 0.036); RPE 11 (D), OTG > ETG (*p* = 0.005) and CG (*p* = 0.006); RPE 13 (E), OTG > ETG (*p* = 0.034), RTG (*p* = 0.007), and CG (*p* = 0.001); RPE 15 (F), OTG > RTG (*p* = 0.018) and CG (*p* = 0.002), ETG > CG (*p* = 0.042); RPE 17 (G), OTG > ETG (*p* = 0.002), RTG (*p* = 0.007), and CG (*p* = 0.001), ETG > CG (*p* = 0.001); RPE 19 (H), OTG > CG (*p* = 0.011), ETG > CG (*p* = 0.008); RPE 20 (I), OTG > CG (*p* = 0.008), ETG > CG (*p* = 0.024). Main effect of group: *F*(3, 92) = 19.84, *p* = 0.001, *η* ^2^ = 0.067. OTG > ETG, RTG, and CG (all *p* = 0.001); ETG > CG (*p* = 0.001); and RTG > CG (*p* = 0.001). Main effect of time: *F*(8, 736) = 236.427, *p* = 0.001, *η* ^2^ = 0.696. Stages 6–9 (RPE 15–20) were significantly higher than Stages 1–5 (RPE 6–13) (*p* < 0.05). Data are presented as mean ± SD (*n* = 24 per group). *p < 0.05; **p < 0.005 (Bonferroni‐corrected). CG, control group; ETG, endurance training group; OTG, open‐skill training group; RPE, rating of perceived exertion; RTG, resistance training group.

According to the two‐way ANOVA results for HbO concentration in the PFC (Figure 5), as shown in Figure 5A–I, there was a significant group‐by‐time interaction during the VO_2max_ test (*F*_group×time_ = 2.464, *P*_group×time_ = 0.001, *η* ^2^ = 0.067). Specifically, as shown in Figure 5C, the HbO of PFC concentration in the OTG was significantly higher than that in the CG (*p* = 0.036). In Figure 5D, the OTG exhibited significantly higher PFC HbO levels than the ETG (*p* = 0.005) and CG (*p* = 0.006). In Figure 5E, the OTG showed significantly higher HbO concentrations than the ETG (*p* = 0.034), RTG (*p* = 0.007), and CG (*p* = 0.001). In Figure 5F, the OTG demonstrated significantly higher PFC HbO levels than the RTG (*p* = 0.018) and CG (*p* = 0.002), whereas the ETG also showed significantly higher PFC HbO concentrations than the CG (*p* = 0.042). As shown in Figure 5G, the OTG displayed significantly higher the HbO of PFC levels than the ETG (*p* = 0.002), RTG (*p* = 0.007), and CG (*p* = 0.001), and the ETG also exhibited significantly higher the HbO of PFC concentrations than the CG (*p* = 0.001). Similarly, in Figure 5H, the OTG had significantly higher the HbO of PFC levels than the CG (*p* = 0.011), whereas the ETG showed significantly higher HbO levels than the CG (*p* = 0.008, *p* = 0.016). In Figure 5I, both the OTG (*p* = 0.008) and ETG (*p* = 0.024, *p* = 0.002) exhibited significantly higher the HbO of PFC concentrations than the CG (*p* < 0.05). The overall mean HbO concentration in the PFC also showed a significant group effect (*F*_group_ = 19.84, *P*_group_ = 0.001, *η* ^2^ = 0.067), reaching a moderate effect size (slightly exceeding the 0.06 threshold). Pairwise comparisons with Bonferroni correction indicated that the OTG had significantly higher PFC HbO concentrations than the ETG (*p* = 0.001), RTG (*p* = 0.001), and CG (*p* = 0.001); the ETG showed significantly higher values than the CG (*p* = 0.001); and the RTG also had significantly higher HbO concentrations than the CG (*p* = 0.001) (*p* < 0.05). In addition, there was a significant time effect for PFC HbO concentrations across fatigue stages during the VO_2max_ test (*F*_time_ = 236.427, *P*_time_ = 0.001, *η* ^2^ = 0.696), with all four groups showing significantly higher HbO levels during Stages 6–9 (RPE 15–20) compared with Stages 1–5 (RPE 6–13) (*p* < 0.05).

### 3.5 Changes in HbT Concentration in the PFC During the VO_2max_ Test

**FIGURE 6:**
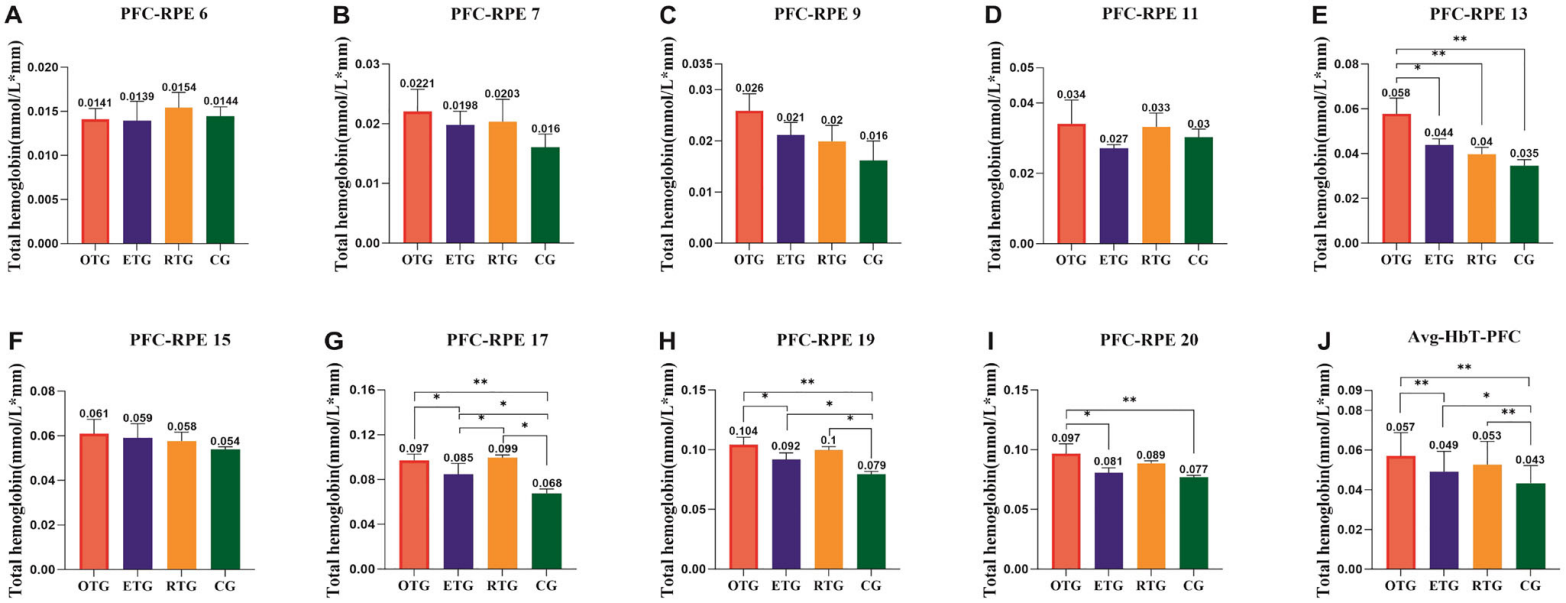
Dynamic changes in total hemoglobin (HbT) concentration in the prefrontal cortex (PFC) during incremental exercise. (A–I) Comparisons of mean HbT concentrations at respective RPE stages among the four groups. (J) Overall mean HbT concentration across all fatigue stages. Statistical analysis: two‐way repeated measures ANOVA (group × time) with Bonferroni post hoc tests. Group × time interaction: *F*(24, 736) = 1.687, *p* = 0.021, *η* ^2^ = 0.047. Significant pairwise differences at specific stages: RPE 13 (E), RTG > ETG (*p* = 0.020), OTG (*p* = 0.003), and CG (*p* = 0.001); RPE 17 (G), OTG > ETG (*p* = 0.041) and CG (*p* = 0.001), RTG > ETG (*p* = 0.015) and CG (*p* = 0.001), ETG > CG (*p* = 0.004); RPE 19 (H), OTG > ETG (*p* = 0.038) and CG (*p* = 0.001), RTG > CG (*p* = 0.042), ETG > CG (*p* = 0.001); RPE 20 (I), OTG > ETG (*p* = 0.008) and CG (*p* = 0.001). Main effect of group: *F*(3, 92) = 16.701, *p* = 0.001, *η* ^2^ = 0.057. OTG > ETG (*p* = 0.001) and CG (*p* = 0.001); ETG > CG (*p* = 0.021); RTG > CG (*p* = 0.001). Main effect of time: *F*(8, 736) = 221.842, *p* = 0.001, *η* ^2^ = 0.682. Stages 5–9 (RPE 13–20) were significantly higher than Stages 1–4 (RPE 6–11) (*p* < 0.05). Data are presented as mean ± SD (*n* = 24 per group). *p < 0.05; **p < 0.005 (Bonferroni‐corrected). CG, control group; ETG, endurance training group; OTG, open‐skill training group; RPE, rating of perceived exertion; RTG, resistance training group.

According to the two‐way ANOVA results for total hemoglobin concentration in the PFC (Figure 6), as shown in Figure 6A–I, there was a significant group‐by‐time interaction during the VO_2max_ test (*F*_group×time_ = 1.687, *P*_group×time_ = 0.021, *η* ^2^ = 0.047). Specifically, as illustrated in Figure 6E, the HbT concentration in the PFC of the RTG was significantly higher than that of the ETG (*p* = 0.020), OTG (*p* = 0.003), and CG (*p* = 0.001). In Figure 6G, the OTG showed significantly higher prefrontal HbT levels than the ETG (*p* = 0.041) and CG (*p* = 0.001), whereas the RTG also exhibited significantly higher values than the ETG (*p* = 0.015) and CG (*p* = 0.001). The ETG demonstrated higher prefrontal HbT concentrations than the CG (*p* = 0.004). As shown in Figure 6H, the OTG had significantly greater HbT levels than the ETG (*p* = 0.038) and CG (*p* = 0.001). Similarly, both the RTG (*p* = 0.042) and ETG (*p* = 0.001) showed higher HbT concentrations than the CG. In Figure 6I, the OTG presented significantly higher prefrontal HbT levels than the ETG (*p* = 0.008) and CG (*p* = 0.001), with statistical significance (*p* < 0.05). The overall mean total hemoglobin concentration in the PFC also showed a significant group effect (*F*_group_ = 16.701, *P*_group_ = 0.001, *η* ^2^ = 0.057), close to a moderate effect size (slightly below 0.06). Pairwise comparisons using the Bonferroni correction indicated that the OTG exhibited significantly higher HbT concentrations than the ETG (*p* = 0.001) and CG (*p* = 0.001). Moreover, the ETG had higher values than the CG (*p* = 0.021), and the RTG also showed significantly greater concentrations than the CG (*p* = 0.001), all reaching statistical significance (*p* < 0.05). In addition, there was a significant time effect for total hemoglobin concentration in the PFC across fatigue stages during the VO_2max_ test (*F*_time_ = 221.842, *P*_time_ = 0.001, *η* ^2^ = 0.682), with all four groups showing higher levels during Stages 5–9 (RPE 13–20) compared with Stages 1–4 (RPE 6–11) (*p* < 0.05).

### 3.6 Changes in FC Strength Between the PFC and MC During the VO_2max_ Test

**FIGURE 7:**
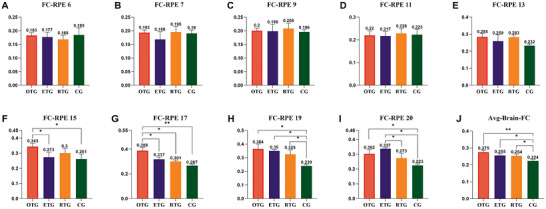
Dynamic changes in functional connectivity (FC) strength between the prefrontal cortex (PFC) and motor cortex (MC) during incremental exercise. (A–I) Comparisons of mean FC strength at respective RPE stages among the four groups. (J) Overall mean FC strength across all fatigue stages. Statistical analysis: two‐way repeated measures ANOVA (group × time) with Bonferroni post hoc tests. Group × time interaction: *F*(24, 736) = 1.57, *p* = 0.04, *η* ^2^ = 0.044. Significant pairwise differences at specific stages: RPE 15 (F), OTG > ETG (*p* = 0.009) and CG (*p* = 0.026); RPE 17 (G), OTG > ETG (*p* = 0.025), RTG (*p* = 0.005), and CG (*p* = 0.001); RPE 19 (H), OTG > CG (*p* = 0.001), ETG > CG (*p* = 0.001), RTG > CG (*p* = 0.006); RPE 20 (I), OTG > CG (*p* = 0.013), ETG > RTG (*p* = 0.044) and CG (*p* = 0.001). Main effect of group: *F*(3, 92) = 8.116, *p* = 0.001, *η* ^2^ = 0.029. OTG > CG (*p* = 0.001); ETG > CG (*p* = 0.018); RTG > CG (*p* = 0.030). Main effect of time: *F*(8, 736) = 25.566, *p* = 0.001, *η* ^2^ = 0.198. Stages 6–9 (RPE 15–20) were significantly higher than Stages 1–4 (RPE 6–11) (*p* < 0.05). Data are presented as mean ± SD (*n* = 24 per group). *p < 0.05; **p < 0.005 (Bonferroni‐corrected). CG, control group; ETG, endurance training group; OTG, open‐skill training group; RPE, rating of perceived exertion; RTG, resistance training group.

According to the two‐way ANOVA results for brain FC strength between the MC and the prefrontal region (Figure 7), as shown in Figure 7A–I, a significant group‐by‐time interaction was observed during the VO_2max_ test (*F*_group×time_ = 1.57, *P*_group×time_ = 0.04, *η* ^2^ = 0.044). Specifically, as illustrated in Figure 7F, the OTG exhibited significantly higher FC strength between the motor and prefrontal regions than the ETG (*p* = 0.009) and CG (*p* = 0.026). In Figure 7G, the OTG showed significantly greater FC strength than the ETG (*p* = 0.025), RTG (*p* = 0.005), and CG (*p* = 0.001). As presented in Figure 7H, the OTG demonstrated significantly stronger FC than the CG (*p* = 0.001), whereas both the ETG (*p* = 0.001) and RTG (*p* = 0.006) also showed significantly higher values than the CG. In Figure 7I, the OTG exhibited greater FC strength than the CG (*p* = 0.013), and the ETG showed higher FC values than both the RTG (*p* = 0.044) and CG (*p* = 0.001), all reaching statistical significance (*p* < 0.05). The overall mean FC strength between the motor and prefrontal regions showed a significant group effect (*F*_group_ = 8.116, *P*_group_ = 0.001, *η* ^2^ = 0.029), corresponding to a small effect size (approximately three times 0.01 but still well below 0.06). Pairwise comparisons with Bonferroni correction indicated that the OTG had significantly higher FC strength than the CG (*p* = 0.001), whereas the ETG (*p* = 0.018) and RTG (*p* = 0.030) also showed higher FC strength compared with the CG (*p* < 0.05). In addition, there was a significant time effect for FC strength between the motor and prefrontal regions across fatigue stages during the VO_2max_ test (*F*_time_ = 25.566, *P*_time_ = 0.001, *η* ^2^ = 0.198), with all four groups showing stronger FC during Stages 6–9 (RPE 15–20) compared with Stages 1–4 (RPE 6–11) (*p* < 0.05).

## 4 Discussion

This cross‐sectional study examined individuals with different training backgrounds (OTG, ETG, RTG, and CG) during an incremental VO_2max_ test, focusing on the dynamic characteristics of PFC activation, motor‐related cortical activation, and their FC, to characterize the association between training background and neural phenotypes under progressive fatigue conditions. The results showed: (1) The VO_2max_ of the OTG and ETG were significantly higher than those of the RTG and CG; (2) the HRV of the OTG was significantly higher than that of the ETG, RTG, and CG. The HRV of the ETG and RTG was also greater than the CG; (3) mean motor cortical HbO did not differ significantly across fatigue stages, but group effects were significant, with OTG showing higher HbO than RTG and CG, and ETG higher than CG. HbO increased markedly from Stages 6–9 (RPE 15–20) compared to Stages 1–5 (RPE 6–13); (4) a significant interaction between fatigue stage and group was observed for motor cortical HbT. Group differences were significant, with OTG showing higher HbT than ETG, RTG, and CG, and ETG higher than CG. HbT increased progressively, with Stages 5–9 (RPE 13–20) higher than Stages 1–4 (RPE 6–11); (5) PFC HbO showed a significant group‐by‐time interaction. OTG had higher HbO than ETG, RTG, and CG, whereas ETG and RTG were higher than CG. HbO rose continuously with fatigue, being significantly higher in Stages 6–9 (RPE 15–20) than in Stages 1–5 (RPE 6–13); (6) PFC HbT also exhibited a significant group‐by‐time interaction. OTG showed higher HbT than ETG and CG, and both ETG and RTG were higher than CG. HbT increased significantly in Stages 5–9 (RPE 13–20) compared with Stages 1–4 (RPE 6–11); and (7) FC between PFC and MC showed a significant group‐by‐time interaction, with overall group differences being significant. OTG demonstrated higher FC than CG, and both ETG and RTG exceeded CG. FC strength increased significantly with fatigue, being higher in Stages 6–9 (RPE 15–20) than in Stages 1–4 (RPE 6–11). However, the observed between‐group differences reflect an association between training background and current neural phenotypes rather than a direct causal effect of training type on CNS function. Given the inherent differences among athletes with different specializations in baseline aerobic capacity, training load, muscle fiber composition, and metabolic characteristics, these confounding factors may interact with training type and jointly shape the observed patterns of cortical activation. Therefore, the following interpretations should be regarded as exploratory explanations of associative patterns, and the causal direction requires further validation through longitudinal randomized controlled intervention studies.

The results of this study indicate significant differences in the performance of participants from different training modalities during the VO_2max_ test. Specifically, the OTG and ETG performed significantly better than the RTG and CG. These findings are consistent with previous research, which suggests that long‐term endurance or open‐skill training can effectively improve an individual's aerobic metabolism and cardiorespiratory function (Gao et al. 2025). Long‐term endurance and open‐skill training are associated with enhanced cardiopulmonary function, blood volume, capillary density, and mitochondrial enzyme activity, which may reflect improved oxygen transport and utilization capacity, and higher VO_2max_ values (Wewege et al. 2018). Resistance training is characterized by high‐intensity anaerobic metabolism …. It is associated with increases in muscle cross‐sectional area and neuromuscular coordination and is linked to relatively smaller VO_2max_ values (Muñoz‐Martínez et al. 2017). Overall, endurance and open‐skill training are more beneficial for improving aerobic capacity and exercise endurance.

HRV is an important physiological indicator for assessing sympathetic and parasympathetic nervous system activity, reflecting the dynamic regulatory capacity of the central autonomic network (CAN) over cardiac rhythm (Hafez and Chang 2025). The results of this study showed that HRV indices improved significantly in all three groups, with open‐skill training demonstrating a clear advantage in improving HRV. This is consistent with previous findings, as endurance training primarily enhances vagal activity, shifting autonomic regulation toward parasympathetic‐dominant homeostasis, whereas resistance training more strongly activates sympathetic output, reflecting short‐term stress adaptations of the cardiovascular system (Stanley et al. 2013). The observed differences may be attributable to autonomic adaptation characteristics associated with different training backgrounds. Notably, the HRV differences observed may be partially explained by confounding factors, such as baseline cardiorespiratory fitness, training load, and metabolic efficiency, rather than being determined solely by training type. Although such training can improve autonomic function, it is insufficient to fully elicit the bidirectional regulatory potential of the central integrative network (Grässler et al. 2021). In comparison, open‐skill training alternates between aerobic and anaerobic components, which is associated with continuous dynamic coordination between the sympathetic and parasympathetic systems and a more flexible autonomic regulatory pattern (Ravier and Marcel‐Millet 2020; Lopez Blanco and Tyler 2025; Tekin et al. 2025). Existing studies have shown that regular open‐skill training not only enhances vagal tone but also promotes functional coupling among key brain regions, such as the PFC, insula, and ACC, thereby optimizing the integrative efficiency of the CAN and enabling more refined control over cardiac rhythm, which is reflected by higher HRV and stronger psychophysiological stability (Butt et al. 2024; Li et al. 2024; Takahashi and Grove 2023). Higher HRV indicates good coordination between central and peripheral systems during autonomic regulation and also reflects the integrative efficiency of the PFC–limbic system in emotional and physiological control, enabling individuals to maintain autonomic stability and functional balance under continuous exercise load or accumulated fatigue, thereby delaying the onset of central and peripheral fatigue; this neuromodulatory pattern may help slow the progression of subjective fatigue perception (Luo et al. 2024; Li et al. 2017; Escorihuela et al. 2020; Schmitt et al. 2015; Hansen et al. 2004).

During the progressive fatigue process, HbO and HbT concentrations in both the motor areas and the PFC increased gradually across all groups. The OTG exhibited the fastest early hemodynamic response and maintained the highest levels throughout the entire protocol, indicating stronger cortical activation and superior cerebrovascular regulation. The ETG showed a stable, steadily rising hemodynamic pattern, whereas the RTG displayed a fluctuating ascending response across the stages of fatigue. The PFC–motor regional FC intensity under OTG was significantly higher than that in other groups. This pattern is consistent with improved cerebral blood supply, oxygen metabolism efficiency, and cross‐regional information integration capacity and may reflect differences in neuromodulation and cognitive mobilization levels. The higher values observed in all training groups relative to the CG align with previous findings, which report that regular exercise is associated with higher levels of molecular factors, such as brain‐derived neurotrophic factor (BDNF), insulin‐like growth factor‐1 (IGF‐1), and vascular endothelial growth factor (VEGF), and with neurotransmitter system modulation that may relate to neurogenesis, synaptic plasticity, and neurovascular coupling. Exercise training is also associated with spinal and central neural network remodeling and gray‐ and white‐matter structural differences, which may relate to brain function and neural health (Marques‐Aleixo et al. 2012; Chen et al. 2025; Tari et al. 2025; Ruiz‐González et al. 2021; Kang et al. 2020).

Differences in neural phenotypes between training groups may be related to long‐term training exposure characteristics specific to different types of sports. Endurance training is associated with cardiorespiratory function, capillary density, and mitochondrial efficiency differences, as well as with subcortical automatic control patterns, lower reliance on cognitive resources, and sustained motor pattern maintenance (Gao et al. 2025; Macías et al. 2022; Mănescu et al. 2026). These characteristics are consistent with an efficient “neural energy‐saving mechanism” and cerebral blood flow regulation and metabolic homeostasis, and the PFC–MC network in endurance athletes shows stable information transfer and synchronization even under high fatigue loads. These patterns may represent training adaptations or preexisting traits (Seifert et al. 2010; Shi et al. 2020). Resistance training is characterized by short‐duration, high‐intensity, intermittent loading, with a focus on muscle strength and neural drive. It is associated with MC excitability and regional blood flow differences but shows limited systemic neurovascular characteristics. Accordingly, cortical activation tends to be transient and spatially localized, and the amplitude of HbO responses during incremental loading is comparatively lower (Herold et al. 2019; Arazi et al. 2021). Open‐skill training involves endurance and explosive components, and athletes in this category engage in continuous behavior regulation in dynamically changing environments, including decision‐making, motor execution, and social interaction (Yin et al. 2025). This type of training is associated with efficient central processing of motor‐execution and cognitive‐control networks that may facilitate coping with complex and evolving contexts.

We propose that the observed cortical activation advantages associated with open‐skill training may be tentatively linked to mechanisms previously reported in the literature, including the upregulation of BDNF and IGF‐1, as well as the potential roles of these factors in synaptic plasticity and white‐matter myelination (Behrendt et al. 2021). However, it is critical to emphasize that these molecular pathways were not directly assessed in the present study; therefore, this interpretation remains speculative and serves as a hypothesis‐generating framework rather than a data‐driven, evidence‐based conclusion. Similarly, the potential contribution of nitric oxide (NO)‐mediated cerebrovascular reactivity and neurovascular coupling, which are hypothesized to arise from intermittent sprint‐induced vascular shear stress, cannot be confirmed by the current fNIRS measurements and should be regarded as a plausible but unverified explanatory model (Mănescu et al. 2026; Green et al. 2012; Chen et al. 2024; Tarumi and Zhang 2015). At the functional level, the stronger PFC–MC coordinated activation observed in the OTG is consistent with the cognitive–motor integration demands of open‐skill training (Strick et al. 2021), though the underlying molecular and vascular substrates require direct empirical validation in future studies. In summary, based on the observed hemodynamic and FC patterns, we hypothesize that open‐skill training may engage vascular regulation, metabolic adaptation, neuroplasticity, and cognitive control mechanisms, potentially resulting in a cortical activation pattern consistent with systemic neural functional optimization. This integrative interpretation, while grounded in existing literature, extends beyond the direct evidence provided by our fNIRS data. The proposed mechanistic links—particularly those involving molecular factors (BDNF, IGF‐1, and VEGF), angiogenesis, and myelination—should be treated as working hypotheses to be tested in future research employing multimodal neuroimaging, blood biomarker analysis, and longitudinal intervention designs.

Overall, changes in cortical activation are jointly constrained by cognitive control demands and the neural metabolic capacity for energy supply. During low‐to‐moderate exercise intensities, increasing demands on executive processing and attention lead to sustained recruitment of cortical neural activity, resulting in a progressive rise in activation levels. However, once exercise intensity exceeds the metabolic supply threshold, oxygen delivery and energy regulation become increasingly limited, leading to disturbances in cerebral hemodynamics and a subsequent suppression of cortical activation. Previous studies have shown that excessive ventilation during high‐intensity exercise reduces arterial partial pressure of carbon dioxide (PaCO_2_), inducing hypocapnia. This reduction in CO_2_ not only inhibits NO synthesis and elevates blood pH but also promotes the release of vasoconstrictive factors, causing contraction of cerebrovascular smooth muscle and a consequent decline in cerebral blood flow (Sato et al. 2016). The resulting limitation in oxygen supply decreases cortical oxygenation, manifested as simultaneous reductions in HbO and HbT. Therefore, cortical activation exhibits a characteristic “inverted‐U” pattern across increasing exercise intensities, initially rising and subsequently declining as fatigue develops (van Duinen et al. 2007; Shigihara et al. 2013; Caldwell et al. 2020).

## 5 Conclusion

This cross‐sectional study compared brain functional differences among athletes with different sport specializations during progressive fatigue, revealing associative characteristics between training background and neural phenotypes related to central regulation. The results showed that participants with open‐skill training and endurance training backgrounds exhibited higher VO_2max_ and HRV, along with distinct cortical hemodynamic profiles. Compared with the ETG, RTG and OTG demonstrated greater prefrontal cortical activation and more pronounced prefrontal–motor cortical FC during moderate‐to‐high fatigue stages. This pattern may be associated with differences in neural activity related to cognitive–motor integration.

Future studies should employ longitudinal intervention designs and randomized controlled trials to further investigate the causal effects of different training modalities on adaptations to central fatigue, while controlling for potential confounders such as training load, metabolic characteristics, and baseline aerobic capacity.

## 6 Limitations

The participants in this study were primarily university athletes aged 19–26, resulting in a relatively narrow age range. Consequently, the generalizability of the findings to other age groups may be limited. Future research could include a broader age range to evaluate the external validity of the conclusions and explore the potential influence of age on neurophysiological adaptations. Furthermore, the cross‐sectional design of this study does not allow for the examination of the temporal dynamics of neural adaptation during training. Longitudinal studies are needed to more comprehensively track the neural adaptation processes of different training modalities over time. The limitations of fNIRS should be explicitly acknowledged: (1) motion artifacts: During the cycling ergometer protocol, 12.3% of channels were excluded at RPE ≥17 due to poor signal quality, limiting the representativeness of data under high‐fatigue conditions; (2) depth limitation: fNIRS only captures cortical activity within approximately 2–3 cm of the surface, whereas subcortical structures, such as the basal ganglia, cerebellum, and brainstem autonomic centers, cannot be assessed; and (3) signal contamination: heart rate‐related scalp blood flow changes may confound neural activation signals. Although short‐separation channel regression was applied for correction, complete separation of systemic and neural components requires integration with modalities such as transcranial Doppler or diffuse correlation spectroscopy. Future multimodal integration is, therefore, essential to validate cortico–subcortical coupling mechanisms. This multimodal fusion approach would provide a more comprehensive exploration of the effects of athletic training on brain network structure, functional integration, and neural plasticity.

## Author Contributions

**Zhi Liu**: conceptualization, data curation, formal analysis, methodology, writing – original draft. **Luxiang Cui**: data curation, formal analysis, writing – review and editing. **Xiaoqi Lu**: conceptualization, formal analysis, methodology, writing – review and editing. **Yangyang Dong**: conceptualization, data curation, formal analysis, methodology, writing – original draft. **Shengting Zhao**: writing – review and editing.

## Funding

This research was supported by the Kunming Municipal Health Science and Technology Talent Training Program (2024‐SW(Reserve)‐97).

## Ethics Statement

The studies involving humans were approved by Ethics Committee of Harbin Sports University (Approval No. 2025035). The studies were conducted in accordance with the local legislation and institutional requirements.

## Consent

The participants provided their written informed consent to participate in this study.

## Conflicts of Interest

The authors declare no conflicts of interest.

## Supporting information




**Supplementary Information**: brb371631‐sup‐0001‐SuppMat.pdf

## Data Availability

The data analyzed in this study is subject to the following licenses/restrictions: The data that support the findings of this study are available on request from the corresponding author. The data are not publicly available due to privacy or ethical restrictions. Requests to access these datasets should be directed to zhaoshengting2026@163.com.
