# Supplementary material for: Dynamic Changes in Cortical Activation Patterns During Incremental Load Among Athletes of Different Sports Types
Source: Brain Behav. 2026 Jul 24;16(7):e71631. doi: 10.1002/brb3.71631 (PMC13397001; doi:10.1002/brb3.71631)
Supplement: Supplementary file 1 — Supplementary Information: brb371631‐sup‐0001‐SuppMat.pdf [file BRB3-16-e71631-s001.pdf]

Supplementary Material

Appendix 1. Method for Determining Sample Size

The sample size in this study was determined using the software G*Power 3.1. According to Cohen's standard, a medium effect size (Cohen's $f = 0.25$) was assumed for the calculation. The significance level was set at $\alpha = 0.05$, and the statistical power ($1-\beta$) was set at 0.80. The number of groups was four. The correlation among repeated measurements was set to the default value of 0.5, and the nonsphericity correction coefficient ε was set to 1. The calculation indicated that twenty participants were required. Considering a possible dropout rate or invalid data of approximately fifteen percent during the study, the final sample size was determined to be twenty-four participants in each group, resulting in a total of ninety-six participants.

Appendix 2. Method for Processing Functional Near-Infrared Spectroscopy Data

The functional near-infrared spectroscopy data were processed using the NirSpark system. First, motion artifacts that were not related to the experimental data were removed. A band-pass filter was then applied to remove components with frequencies higher than 0.1 hertz and lower than 0.01 hertz, in order to eliminate physiological noise such as cardiac and respiratory signals and to reduce baseline drifts caused by environmental and temperature variations.

The collected optical signals were processed according to the modified Beer–Lambert law to obtain hemodynamic data related to blood oxygenation. Subsequently, the Network module of the NirSpark software was used to calculate the changes in blood oxygen concentration at each time point during the maximal oxygen uptake test. Pearson correlation coefficients were computed for the time-series data of oxygenation signals between two regions of interest. These correlation coefficients were then converted using Fisher's Z-transformation, and the transformed values were defined as the strength of functional connectivity between channels as well as the mean value of cortical functional connectivity.
